# Prostate-specific membrane antigen (PSMA) expression in adenoid cystic carcinoma of the head and neck

**DOI:** 10.1186/s12885-020-06847-9

**Published:** 2020-06-05

**Authors:** Thomas J. W. Klein Nulent, Matthijs H. Valstar, Laura A. Smit, Ludwig E. Smeele, Nicolaas P. A. Zuithoff, Bart de Keizer, Remco de Bree, Robert J. J. van Es, Stefan M. Willems

**Affiliations:** 1 Department of Head and Neck Surgical Oncology, UMC Utrecht Cancer Center, University Medical Center Utrecht, Heidelberglaan 100, P.O. Box 85500, Utrecht, 3508 GA The Netherlands; 2 Department of Oral and Maxillofacial Surgery, University Medical Center Utrecht, Utrecht, The Netherlands; 3 Department of Head and Neck Oncology and Surgery, Netherlands Cancer Institute/Antoni van Leeuwenhoek, Amsterdam, The Netherlands; 4 Department of Oral and Maxillofacial Surgery, Amsterdam UMC, University of Amsterdam, Amsterdam, The Netherlands; 5 Department of Pathology, Netherlands Cancer Institute/Antoni van Leeuwenhoek, Amsterdam, The Netherlands; 6 Julius Center for Health Sciences and Primary Care, University Medical Center Utrecht, Utrecht, The Netherlands; 7 Department of Radiology and Nuclear Medicine, University Medical Center Utrecht, Utrecht, The Netherlands; 8 Department of Pathology, University Medical Center Utrecht, Utrecht, The Netherlands

**Keywords:** Adenoid cystic carcinoma, Salivary gland neoplasms, Immunohistochemistry, Survival analysis, PSMA, Prostate-specific membrane antigen

## Abstract

**Background:**

Treatment options for advanced head and neck adenoid cystic carcinoma (AdCC) are limited. Prostate-Specific Membrane Antigen (PSMA), a transmembrane protein that is known for its use in diagnostics and targeted therapy in prostate cancer, is also expressed by AdCC. This study aimed to analyse PSMA expression in a large cohort of primary, recurrent and metastasized AdCC of the head and neck.

**Methods:**

One hundred ten consecutive patients with histologically confirmed AdCC in the period 1990–2017 were included. An analysis was made of clinical details, revised pathology and semiquantitative immunohistochemical expression of PSMA on tissue microarray and whole slides. Associations of PSMA expression with clinicopathological parameters were explored and survival was analysed by multivariate Cox-proportional Hazard analysis.

**Results:**

PSMA expression was present in 94% of the 110 primary tumours, with a median of 31% positive cells (IQR 15–60%). Primary tumours (*n* = 18) that recurred (*n* = 15) and/or had metastases (*n* = 10) demonstrated 40, 60 and 23% expression respectively. Expression was not independently related to increased pathological stage, tumour grade, and the occurrence of locoregional recurrence or metastasis. After dichotomization, only primary tumour PSMA expression ≤10% appeared to be associated with reduced 10-years recurrence-free survival (HR 3.0, 95% CI 1.1–8.5, *p* = .04).

**Conclusions:**

PSMA is highly expressed in primary, recurrent and metastatic AdCC of the salivary and seromucous glands. PSMA expression has no value in predicting clinical behaviour of AdCC although low expression may indicate a reduced recurrence-free survival. This study provides supporting results to consider using PSMA as target for imaging and therapy when other diagnostic and palliative treatment options fail.

## Background

The Prostate-specific membrane antigen (PSMA) is a transmembrane glycoprotein of the prostate secretory acinar epithelium that is upregulated in prostate cancer (PC) and known from its use in diagnostics and targeted therapy in metastatic PC [1–4]. Besides tracer accumulation in prostate tissue, PSMA PET/CT depicts physiological uptake in the salivary and lacrimal glands, liver and kidneys, but also in benign and malignant neoplasms, mostly adenomas and (adeno) carcinomas, of glandular or epithelial origin [5, 6].

In PC, increased intracellular PSMA expression by immunohistochemistry is related to increased pathological grade, and subsequently correlated with disease-related mortality [1–4]. Malignancies other than PC also express PSMA but in endothelial cells of tumours’ neovasculature, which suggests PSMA involvement in tumour angiogenesis. In salivary glands PSMA was identified on the acinar cells in the epithelium [3, 7–9].

Recently, PSMA PET/CT analysis in a series of patients with head and neck adenoid cystic carcinoma (AdCC) showed tracer uptake in areas of locoregional recurrent and distant metastatic AdCC, and expression was confirmed immunohistochemically [10]. AdCC is the most common malignant secretory gland tumour in the head and neck region. Incidence peaks in the fifth and sixth decade and has a female predominance [11–15]. AdCC originates from ductal (luminal) and basal/myoepithelial (abluminal) cells and typically arises in the major salivary glands, the minor salivary and seromucous glands of the lip and upper aerodigestive tract, but also in the lacrimal and ceruminous glands. The tumour is characterized by an indolent but persistent growth rate, frequent locoregional recurrence and a delayed silent onset of distant metastasis, mainly in the lungs [11, 15–17]. Surgery is the primary treatment, frequently followed by adjuvant radiation therapy because of positive resection margins and typical perineural growth. Although radiotherapy has probably no benefit to survival, it is reported to improve local and regional control [15, 16]. Disease-specific survival (DSS) is moderate, with five and 10 year survival rates of 68–78% and 54–65% respectively [18, 19]. Survival is negatively affected by the occurrence of an irresectable locoregional recurrence, which is considered clinically more relevant than the occurrence of slowly growing -often pulmonary and osseous- distant metastases that develop in almost half of the patients within 5 years after diagnosis [11, 16, 18]. Other negative prognostic factors are advanced tumour stage, inadequate resection margins, skull base involvement and a solid growth pattern on histopathology. Perineural invasion does not directly affect mortality, but is significantly correlated with metastatic disease [11, 15]. Regular treatment options are limited in advanced recurrent or metastasized disease [16]. Given the positive results of PSMA-targeted diagnostic and treatment modalities in PC, this study aimed to analyse PSMA expression in a large cohort of primary and corresponding recurrent and metastatic AdCC tissues of the head and neck [20]. Secondly, we aimed to explore associations with patient- and tumour characteristics and outcome, analogous to PC.

## Methods

### Patient selection

All patients diagnosed with AdCC in the University Medical Center Utrecht and Netherlands Cancer Institute-Antoni van Leeuwenhoek Hospital between 1990 and 2017 were analysed in a retrospective cohort study. Patients were selected in case of a histology-proven primary AdCC in the head and neck region with available representative formaldehyde-fixed paraffin-embedded tissue blocks of the resection specimen. Tumour samples of available corresponding locoregional recurrences or distant metastases were collected. Patients with previous (non AdCC) salivary gland disease, radiotherapy to the head or neck, or incomplete data were excluded. All data and samples were handled according to the GDPR.

### Clinical parameters and tumour characteristics

The following clinical parameters were retrieved from the medical files: patient’s gender, age at diagnosis, tumour site, treatment regimen, (time to) recurrence or metastasis, vital status (cause of death) and date of last follow-up until January 1st 2018. Two dedicated head and neck pathologists (S.W. and L.S.) re-examined all haematoxylin- and eosin-stained slides for the following parameters: type and diameter of the tumour, pathological T- and N-stage, histopathological growth pattern and associated grade according to the differentiation of Perzin et al. [21], surgical resection margins and the presence of perineural, vascular and bone invasion.

### PSMA immunohistochemistry

A Tissue Microarray (TMA) was used to assess PSMA expression. From each tumour, three central 0.6 mm tissue cylinders from vital tumour were incorporated and covered the different aspects of this tumour morphology. Tumour whole-slides were analysed when patient’s tissue was not incorporated in the microarray, as well as whole-slides of all available recurrent and distant tissues. Representative TMA or whole slide paraffin sections 4 μm thick were immunohistochemically stained using fully automated protocols on the Benchmark XT (Ventana Medical Systems, Tucson, AZ, USA), validated for diagnostic purposes. Incorporated as control tissues were prostate cancer, normal salivary gland and duct tissue. For the primary antibody, a mouse antihuman PSMA monoclonal antibody was used (3E6; DAKO, Carpinteria, CA) of the IgG1 isotype directed against the internal domain of the PSMA antigen (DAKO, cat. no. M3620, Carpinteria, CA, dilution 1/80). The tissue sections were deparaffinised with xylene and ethanol followed by Heat Induced Epitope Retrieval in Ventana Cell Conditioning 1 for 24 min and subsequently incubated with the primary antibody for 60 min. Antigen-antibody reactions were visualized using Ventana OptiViewTM Amplification kit, followed by Ventana OptiViewTM Universal DAB Detection Kit (Optiview HQ Linker 8 min, Optiview HRP Multimer 8 min, Optiview Amplifier H2O2/Amplifier 4 min, Optiview Amplifier Multimer 4 min). Finally the slides were counterstained with haematoxylin, dehydrated and mounted.

### PSMA expression analysis

Blinded semiquantitative scoring of all selected primary, recurrent and distant AdCC tumour samples was done until consensus was reached by two head and neck pathologists and two researchers (S.W., L.S., T.K.N. and M.V.). Per tumour core or whole slide the localization of PSMA-positive tumour cells was noted, followed by scoring the percentage of positive tumour cells in increments of 5%. Total tumour PSMA expression of the arrayed cores was defined by the mean percentage of PSMA-positive tumour cells of the three tissue cores. A core was considered inadequate when it contained < 5% tumour tissue. In case of a mean PSMA expression below 10% or in case of more than 1 inadequate core on microarray, one representative tumour whole slide was subsequently stained and scored in order to exclude false-negative results.

### Statistical evaluation

Continuous and ordinal variables were reported as medians with interquartile ranges (IQR), categorical variables were reported as the number of patients and percentages. Associations between PSMA expression and all clinical parameters and tumour characteristics, except when categorical with more than 3 categories, were estimated with Spearman-ρ correlation coefficient with corresponding *p*-values. The Independent Samples Kruskal-Wallis test (KW) was used to compare expression distribution between patients’ primary tumours, which recurred or metastasized and those that did not. Subsequently, the median expression of corresponding primary, recurrent and distant samples was compared. A cut-off level for the prediction of overall survival (OS), DSS, locoregional recurrence-free survival (RFS), and metastatic-free survival (MFS) was determined by dichotomizing PSMA expression and plotting Receiver Operating Characteristic (ROC)-curves. Differences in baseline characteristics of the groups divided by dichotomization were compared using Pearson chi-square test with appropriate Bonferroni correction. Statistics were performed using SPSS Statistics (version 22.0, IBM Corp., Armonk, NY, USA) for Windows. Multivariate OS, DSS, RFS and MFS survival analyses were carried out to calculate Hazard ratios with 95% confidence interval (CI). A Cox-proportional Hazard regression model was created by using SAS software (version 9.4, SAS Institute Inc., Cary, NC, USA) for Windows. Firth’s correction was applied to reduce bias of maximum likelihood estimation, as it deals with the occurrence of monotone likelihood in small-sample studies with time-dependent effects [22]. Discriminative ability of the model was assessed by computing Harrell’s C-statistic [23]. A two-tailed *P-*value < 0.05 was considered statistically significant for all analyses.

## Results

### Patients, clinical parameters and tumour characteristics

In total 122 newly diagnosed patients with AdCC of the head and neck were identified within the 27-year period, of whom 12 were excluded because of incomplete data. From 110 patients the clinical history and histopathological samples of the primary tumour were available for analysis and summarized in Table 1.

**Table 1:** Cohort Characteristics

	N (%)	Median % PSMA	Spearman-ρ; *p*-value	PSMA≤10%	PSMA>10%	Chi^**2**^ PSMA ≤10% vs >10%
**Patients**	110	31%		23 (21%)	87 (79%)	
Gender						
Male	36 (33%)	30%	.11; *p* = .24	7 (19%)	29 (81%)	*p* = .79
Female	74 (67%)	45%		16 (22%)	58 (78%)	
Age at diagnosis						
Median (IQR)	57 (45–68)		.08; *p* = .42	56 (50–70)	58 (43–68)	*p* = .45
Range	20–90			34–87	20–90	
Site and subsite						
^a^Major salivary gland	59 (54%)	50%	−.25; *p* < .01	7 (12%)	52 (88%)	*p* = .01
Parotid gland	26	50%		2	24	*p* = .06
Submandibular gland	30	35%		5	25	*p* = .50
Sublingual gland	3	57%		0	3	*p* = .37
^a^Minor salivary and seromucous gland	51 (46%)	21%		16 (31%)	35 (69%)	
Oral cavity (lip/buccal mucosa/hard palate/gum)	21	19%		7	14	*p* = .12
Oropharynx (soft palate/base of tongue)	8	21%		1	7	*p* = .54
Nasal cavity/nasopharynx/maxillary sinus	15	10%		8	7	*p* < .01
Larynx/trachea	3	42%		0	3	*p* = .37
Lacrimal gland	2	35%		0	2	*p* = .46
External auditory canal	2	40%		0	2	*p* = .46
**Tumour**						
pT-stage (TNM 7th ed.)						
pT1	35	50%	−.18; *p* = .06	5	30	*p* = .24
pT2	39	33%		7	32	*p* = .57
pT3	5	20%		1	4	*p* = .96
pT4a	23	30%		6	17	*p* = .49
pT4b	8	13%		4	4	*p* = .04
Nodal status						
pN0	99 (90%)	33%	−.09; *p* = .37	20 (20%)	79 (80%)	*p* = .58
pN+	11 (10%)	25%		3 (27%)	8 (73%)	
Distant metastasis						
cM0	108	31%	−.01; *p* = .90	23 (21%)	85 (79%)	*p* = .62
cM1	2	36%		0 (0%)	2 (100%)	
Resection margin						
clear (> 5 mm)	20 (18%)	50%	−.08; *p* = .43	2 (10%)	18 (90%)	*p* = .18
close (1-5 mm)	4 (4%)	20%		2 (50%)	2 (50%)	*p* = .14
positive (< 1 mm)	86 (78%)	30%		19 (22%)	67 (78%)	*p* = .56
Perineural growth						
Present	76 (69%)	30%	−.02; *p* = .86	14 (18%)	62 (82%)	*p* = .44
Absent	32 (31%)	45%		8 (25%)	24 (75%)	
Vasoinvasive growth						
Present	17 (15%)	30%	−.05; *p* = .64	5 (29%)	12 (71%)	*p* = .33
Absent	90 (82%)	33%		17 (19%)	73 (81%)	
^a^Bone invasion						
Present	27 (25%)	17%	−.20; *p* = .04	10 (37%)	17 (63%)	*p* = .02
Absent	82 (75%)	39%		13 (16%)	69 (84%)	
Growth pattern (Perzin grade [21])						
Tubular (grade 1)	46 (42%)	30%	.12; *p* = .21	10 (22%)	36 (78%)	*p* = .89
Cribriform; < 30% solid (grade 2)	43 (39%)	33%		7 (16%)	36 (84%)	*p* = .32
Solid (grade 3)	20 (18%)	46%		6 (30%)	14 (70%)	*p* = .28
**Treatment**						
Adjuvant radiotherapy						
Yes	102 (93%)	33%	.14; *p* = .15	20 (20%)	82 (80%)	*p* = .23
No	8 (7%)	26%		3 (38%)	5 (62%)	

^a^ difference is considered statistically significant

### PSMA immunohistochemistry

**Fig. 1:**
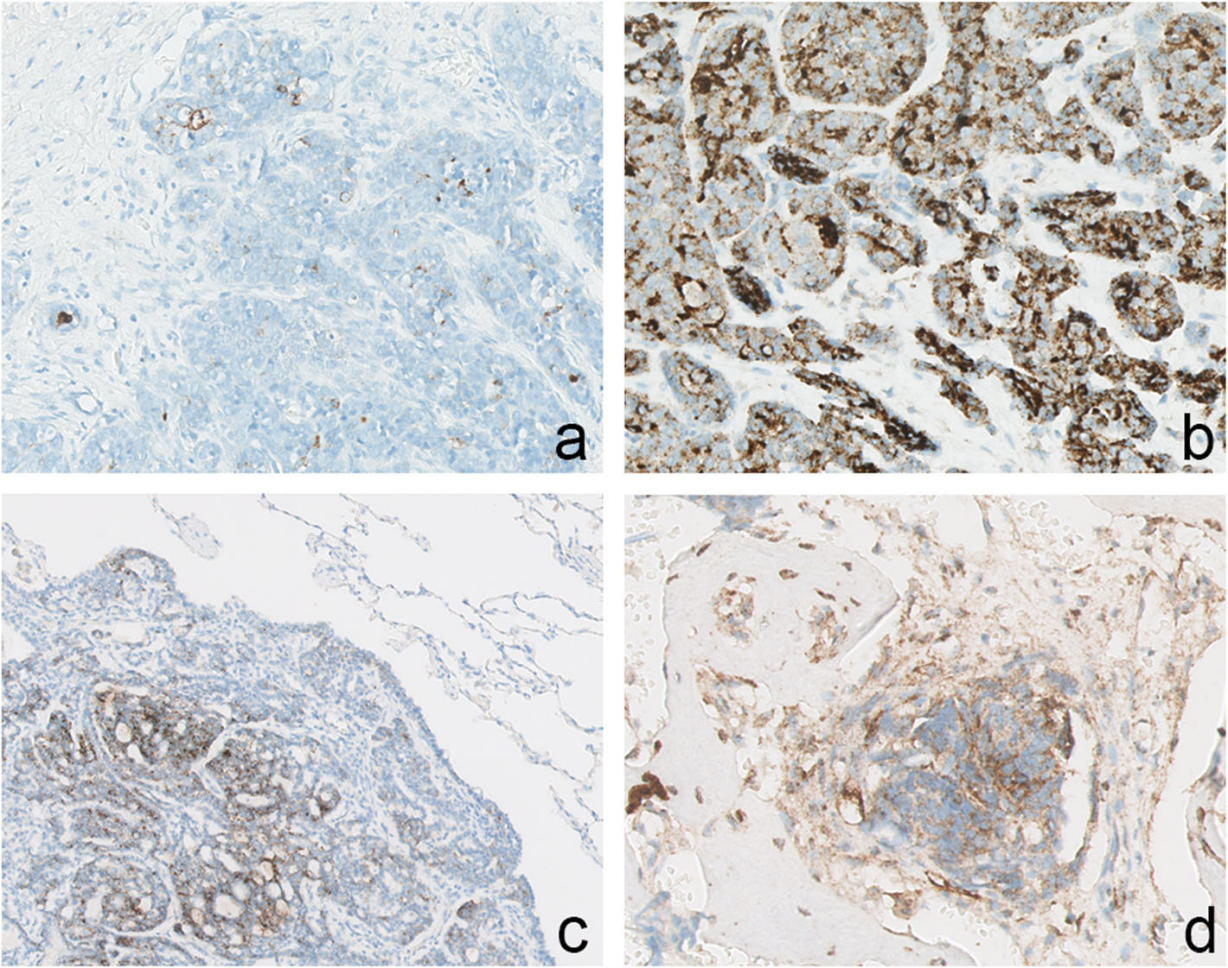
Representative immunohistochemical PSMA expression in primary and metastatic AdCC showing variation in the number of positive staining tumour cells. **a** nasal cavity; 5%; *magnification 200x.***b** submandibular gland; 90%; *magnification 400x.***c** pulmonary metastasis; 70%; *magnification 200x.***d** bone metastasis; 5%; *magnification 200x*

Samples of 73 out of 110 patients were available for TMA immunohistochemistry. Of the remaining 37 primary tumours and available locoregional (*n* = 15) and distant (*n* = 10) tumour samples whole-slides were separately stained and scored. Expression was seen intracellular in a granular fashion, mainly cytoplasmic, or concentrated at the luminal side of the cell membrane. No conclusive staining pattern within the cells was observed. The staining intensity was consistent and there was limited spatial variability. Staining of the whole slides and microarray cores were comparable and therefore usage of this TMA was considered reliable. TMA analysis of 14 tumours was unsuccessful due to inadequate cores, and 21 tumours initially scored 0–10% expression. Matched whole slides of these 35 tumours were subsequently stained and PSMA expression was adjusted in six cases with conflicting (> 10% difference) results. Different immunohistochemistry examples are visualized in Fig. 1.

### PSMA expression analysis in primary AdCC

In 103 out of 110 primary tumours (94%) PSMA was expressed with median 31% positive tumour cells (IQR 15–60%). Median PSMA expression and Spearman-ρ correlation coefficients for all clinical parameters and pathological characteristics are listed in Table 1. Although no strong associations were found, Spearman-ρ analysis revealed a significant association between PSMA expression and both tumour subsite and bone invasion. The minor salivary glands demonstrated less PSMA expression than the major salivary glands (21 vs. 50% median expression; *p* < .01) and tumours invading bone showed less PSMA expression (17 vs. 39% median expression; *p* = .04). However, tumour subsite and bone invasion are mutually strongly correlated (Pearson chi-square test *p <* .01) as all except 1 of the tumours invading bone originated from the minor salivary glands. Median PSMA expression did not differ between the subgroups of tumour site, pathological tumour stage (pT-stage) and Perzin grade. Furthermore, primary tumour PSMA expression medians of patients who developed a locoregional recurrence or distant metastasis seem higher, but did not differ statistically from the distribution of those who remained disease-free (Table 2).

**Table 2:** Follow-up

	N (%)	Median months; IQR	Median % PSMA primary tumour	PSMA 0–10%	PSMA >10%	Chi^**2**^ PSMA ≤10% vs >10%
Patients	110			23	87	
**Follow-up** (median months; IQR)		57; 26–100		50; 13–96	63; 27–110	
Locoregional recurrence						
Yes	29 (26%)	39; 20–91	40%	9	20	*p* = .12
No	81 (74%)		30%	14	67	
Distant metastasis						
Yes	36 (33%)	33; 12–77	38%	7	29	*p* = .79
No	74 (67%)		30%	16	58	
^a^Locoregional recurrence-free @ 10 years						
Yes	84 (76%)		35%	14	70	*p* = .05
No	26 (24%)		31%	9	17	

^a^ difference is considered statistically significant

### PSMA expression analysis in recurrent and distant metastatic AdCC

Fifteen locoregional recurrent and 10 distant metastatic tissue samples could be retrieved from 18 patients. Nine of these 18 patients developed only locoregional recurrence(s), 6 patients developed only distant metastases and 3 patients developed both a recurrence and metastases. Distant metastatic tissues were lung (3 cases), leptomeningeal (3 cases), bone (1 case), liver (1 case), peritoneum (1 case) and skin (1 case). Positive PSMA expression was seen in 12/15 (80%) recurrent and 9/10 (90%) distant samples that ranged from 5 to 100%. All together, the median PSMA expression of the recurrent samples was 60% (IQR 30–90%) and of all metastatic samples 23% (IQR 10–55%). When these were compared to the expression of their corresponding 18 primary tumours (40% median expression; IQR 15–70%), there was a (non-significant) tendency of increased expression in recurrences and decreased expression in distant metastases. Expression patterns of corresponding tumour samples are visualized in Supplementary Figure 1.

### Follow-up and survival analysis

Details of follow-up and survival rates are summarized in Tables 2 and 3. Dichotomization of PSMA expression was carried out by plotting different ROC-curves for prediction of OS, DSS, RFS and MFS, which showed optimal cut-off points in a range of 8–12%. Although the area under these curves was not sufficient to assume high sensitivity and specificity, for practical reasons a merged cut-off point of 10% PSMA expression was defined (graphs not shown). The 0–10% expression group was dominated by patients whose tumour was located in the minor salivary glands and, more specifically, in the nasal cavity, nasopharynx or maxillary sinus as revealed by post-hoc analysis (*p* = .01 and *p* < .01 respectively). Furthermore, in this group, relatively more tumours showed bone invasion (*p* = .02) and relatively more patients were not recurrence-free 10 years after diagnosis (*p* = .05). Despite half of the pT4b tumours expressed 0–10% PSMA, this distribution did not reach statistical significance due to multiple testing (*p* = .04), see Table 1. A multivariate Cox-proportional Hazard model with Firth’s correction showed a significant relation between low PSMA expression (0–10%) and worse 10-years RFS (HR 3.0, 95% CI 1.1–8.5, *p* = .04). A > 30% solid growth pattern / Perzin grade 3 (HR 3.7, 95% CI 1.3–11.2, *p* = .02), tumour localization in the nasal cavity, nasopharynx or maxillary sinus (HR 41.7, 95% CI 6.2–236.8, *p* < .01), and no postoperative radiotherapy (HR 5.1, 95% CI 1.2–17.5, *p* = .02) were also significant prognosticators for locoregional recurrence within 10 years. Harrell’s C-statistic of the predictive Cox-proportional Hazard model was 0.81, and without PSMA expression 0.79. PSMA expression was no significant contributor for the prediction of OS, DSS and MFS (multivariate data not shown).

**Table 3:** Survival data

	N affected	Median months	% survival
Overall survival			
5-year OS	25	36	77%
10-year OS	29	41	74%
Disease-specific survival			
5-year DSS	17	41	85%
10-year DSS	21	47	81%
Disease-free survival			
5-year DFS	36	20	67%
10-year DFS	49	31	55%
Locoregional recurrence-free survival			
5-year RFS	19	27	83%
10-year RFS	26	36	76%
Metastatic-free survival			
5-year MFS	26	22	76%
10-year MFS	33	28	70%

## Discussion

This is the first large cohort study describing PSMA expression in primary, recurrent and distant metastatic AdCC of the head and neck. Positive expression was seen in 94% of the primary tumours, 80% of recurrent tumours and 90% of the distant metastases, with PSMA expressed in 31, 60 and 23% of the tumour cells respectively. Primary tumour expression could not indicate disease progression and could not estimate expression levels in a recurrence or metastasis, although a tendency of respectively increase and decrease was observed. PSMA expression was not correlated to pathological stage and grade.

This is in contrast to PC in which high PSMA expression is correlated with prostate-specific antigen (PSA) recurrence and other prognostic factors which negatively affect survival such as tumour grade, pathological stage and castration resistance [4, 24]. Multiple studies have shown that PSMA activates AKT and MAPK pathways promoting proliferation and survival of cancer cells, which may lead to an aggressive biological and clinical behaviour [25, 26]. However, the currently presented inverse correlation of low primary tumour PSMA expression ≤10% as independent predictor of shortened RFS (HR 3.0; 95% CI 1.1–8.5; *p* = .04) has also been described in other cancer types and might partly be explained by epigenetic silencing of the PSMA gene upon tumour progression [27].

Analogous to PC, AdCC demonstrates expression of PSMA in the epithelial tumour cells, while expression in different other tumours mainly concentrates in endothelial cells of tumour-associated neovasculature [3, 6, 28].

Of all primary PCs, 95% show heterogeneous PSMA expression with on average 53 ± 32% (mean ± SD) positive tumour cells. Mean expression in regional lymph nodes and distant metastasis is more extensive (72 ± 36% and 92 ± 10% respectively). Normal prostate tissue shows high PSMA expression in 100% of the samples (77 ± 32% positive cells), but with significantly less staining intensity than tumour tissue [3, 4].

In contrast, staining intensity in AdCC is relatively constant. Although it is known that major and minor salivary glands depict high tracer uptake on PSMA PET/CT, a comparison between expression intensity in normal salivary gland tissue and AdCC tumour tissue could not be made due to the lack of data on PSMA expression in non-pathologic salivary glands [29]. Comparing published PC data and our AdCC data, the present study concludes that similar to PC, 94% of primary AdCC expresses PSMA, but AdCC expression is more homogenous in a lower percentage of positive tumour cells.

Some points need to be addressed. Multiple Spearman-ρ correlation analyses and KW nonparametric tests were carried out to analyse possible differences between a large amount of clinical parameters and tumour characteristics. False-positive findings might have been introduced, as adjustments to correct for multiple comparisons are not desirable in explorative studies and were therefore not applied. The results of these analyses should therefore be interpreted carefully [30].

By comparing the dichotomized groups of PSMA expression, it is noticed that a relatively large number of tumours in the low ≤10% PSMA expression group are located in the nasal cavity, nasopharynx or maxillary sinus, sites that are known to have a worse prognosis when compared to tumours originating from other subsites [11]. Notwithstanding the small sample sizes in these study subgroups, specifically the aforementioned tumour sites are, besides poor RFS, concordantly associated with poor DSS and OS (data not shown). Tumour stage T4b and bone invasion were also over-represented in the low PSMA expression (≤10%) group. Although these parameters themselves are no independent predictors of RFS, tumour stage is strongly correlated with DSS and OS. Furthermore, tumour stage and bone invasion are mutually highly correlated (Pearson chi-square test *p <* .01) as all except one T4b tumour showed bone invasion. Moreover, bone invasion is significantly associated with tumour localization in the nasal cavity, nasopharynx or maxillary sinus. Tumours at these locations recur more often. These collinearities could be explained by delayed presentation of tumours from this subsite (that often involves the skull base), but may have confounded the results.

Another factor of debate is the limited discriminative strength of the 10% cut-off point. The above mentioned considerations are supported by the minimal increase of Harrell’s C-statistic of the multivariate Cox-proportional Hazard model by 0.02 and therefore the additive value of PSMA to the prediction of RFS remains questionable.

A deep locoregional recurrence or growing distant metastases, for which conventional treatment options are no longer applicable due to functional irresectability or exceeded radiation limits, is a relevant problem in the management of AdCC. The limited experience with palliative chemotherapeutic agents and new initiatives with different targeted agents in this setting was recently reviewed [31]. The present results of high PSMA expression in primary, recurrent and metastatic tumour cells with limited spatial and temporal variability, as well as the uptake of PSMA ligand in recurrent and distant metastatic AdCC on PET/CT, could suggest a potential role for palliative targeted treatment with Lutetium-177-PSMA [10, 32]. First large trials of this radionuclide treatment of metastatic castration-resistant PC show high response rates with low toxicity, improved quality of life and even prolonged OS [20, 33]. Regarding AdCC, the high amount of associated grade I xerostomia (87%) must be taken into account, as these patients usually have already been exposed to radiotherapy to the head and neck before [20].

## Conclusions

This study shows unambiguous PSMA expression in a large cohort of primary, recurrent and metastatic AdCC of the head and neck. Expression was seen in 94% of the primary tumours, which is analogous to PC except for the median lower number of positive tumour cells. In general, there was no relation between upregulated PSMA expression and pathological stage, tumour grade (growth pattern), the occurrence of locoregional recurrence or metastasis, and survival. Low primary tumour expression ≤10% is significantly associated with worsened RFS although its predictive value is limited. Of the recurrent and distant samples, respectively 87 and 90% were PSMA-positive, but staining could not be estimated based on primary tumour expression. This study provides encouraging supporting results that when other palliative systemic treatment options fail, PSMA-targeted imaging followed by experimental Lutetium-177-PSMA radionuclide therapy in AdCC, might be an alternative.

## Supplementary information

**Additional file 1: Supplementary Figure 1.** Differences in PSMA expression between primary, recurrent and metastatic AdCC in 18 patients, in order of PSMA expression of the primary tumour.

## Data Availability

The datasets used and/or analysed during the current study are available from the corresponding author on reasonable request.

## Abbreviations

PSMA: Prostate-Specific Membrane Antigen
AdCC: Adenoid cystic carcinoma
PC: Prostate carcinoma
TMA: Tissue microarray
OS: Overall survival
DSS: Disease-specific survival
DFS: Disease-free survival
RFS: Recurrence-free survival
MFS: Metastatic-free survival
IQR: Interquartile range
CI: Confidence interval
HR: Hazard ratio
KW: Kruskal-Wallis test
ROC: Receiver Operating Characteristic
