# Supplementary material for: Prostate-specific membrane antigen (PSMA) expression in adenoid cystic carcinoma of the head and neck
Source: BMC Cancer. 2020 Jun 5;20:519. doi: 10.1186/s12885-020-06847-9 (PMC7275445; doi:10.1186/s12885-020-06847-9)
Supplement: Supplementary file 1 — Additional file 1: Supplementary Figure 1. Differences in PSMA expression between primary, recurrent and metastatic AdCC in 18 patients, in order of PSMA expression of the primary tumour. [file 12885_2020_6847_MOESM1_ESM.docx]

**Supplementary figure 1:** Differences in PSMA expression between primary, recurrent and metastatic AdCC in 18 patients, in order of PSMA expression of the primary tumour.
